# Tissue-specific DNA repair strategies underlie apparent high transposon activity in the *Caenorhabditis elegans* soma

**DOI:** 10.1093/g3journal/jkag054

**Published:** 2026-03-18

**Authors:** Cindy Chang, David D Lowe, Scott Kennedy

**Affiliations:** Department of Genetics, Harvard Medical School, Boston, MA 02115, United States; Department of Genetics, Harvard Medical School, Boston, MA 02115, United States; Department of Genetics, Harvard Medical School, Boston, MA 02115, United States

**Keywords:** transposon, *Caenorhabditis elegans*, double-strand break, DNA repair, NHEJ

## Abstract

Transposons are parasitic nucleic acids that threaten genome integrity in all organisms. DNA transposons mobilize via a cut-and-paste mechanism, which leads to double-strand breaks (DSBs). In *Caenorhabditis elegans*, DNA transposons are mobile in the soma, where their excision rates are reportedly ≅1,000-fold higher than in germ cells. How or why DNA transposons might be highly active in the *C. elegans* soma is a mystery. Here, we show that the non-homologous end joining (NHEJ) pathway is responsible for generating >99.9% of the empty transposon sites accruing in the *C. elegans* soma. *C. elegans* uses homologous recombination (HR) to repair transposon-induced DSBs in its germline. Because HR, but not NHEJ, restores excised transposons back into their original chromosomal position during repair, we propose that the apparent elevated activity of DNA transposons in the *C. elegans* soma can, in large part, be explained by tissue-specific differences in DNA repair strategy.

## Introduction

Transposons are mobile genetic elements that threaten all eukaryotic genomes. Transposons are classified into 2 classes: The class I retrotransposons, which use an RNA intermediate and reverse transcription to replicate; and the class II DNA transposons, which mobilize via a direct DNA cut-and-paste mechanism (Wicker et al. 2007). Transposons—and the remnants of ancient transposons—constitute between 3% and 85% of eukaryotic genomes (Huang et al. 2012). For example, about 12% of the genome of the metazoan model organism *Caenorhabditis elegans* is composed of transposons, with the most active and abundant of these being the class II *Tc1*/*Mariner* family DNA transposons (Fischer et al. 2003; Huang et al. 2012). Most active *Tc1*/*Mariner* elements in *C. elegans*, including *Tc1–3* and *Tc5*, produce a single RNA that encodes a transposase enzyme that is responsible for the excision and re-insertion (cut-and-paste) of the element (Plasterk 1996; Fischer et al. 2003). Transposons that mobilize via cut-and-paste can increase copy number within genomes via 2 mechanisms: First, mobilization of a transposon from replicated DNA to non-replicated DNA during mitosis or meiosis can increase transposon copy number (Fedoroff 1989). Second, a DSB caused by transposon excision can be repaired by homologous recombination (HR), using the sister or homologous chromosome as a repair template. When this happens, HR restores the transposon back into the donor site and, if the original transposon inserts elsewhere in the genome, copy number increases (Hagemann and Craig 1993). This latter mechanism of repair by HR appears to operate in the *C. elegans* germline as animals made hemizygous for *Tc1* exhibit a ≅100-fold increase in germline excision rates compared to animals homozygous for the same element (Plasterk 1991). Similar observations have been made in *Drosophila* (Engels et al. 1990). In both cases, it has been suggested that because the template used for repair in hemizygous animals lacks a transposon, HR-based repair cannot restore the transposon into its original position, resulting in transposon excision (Engels et al. 1990; Plasterk 1991). Thus, homology-based DSB repair masks most transposon excision events occurring in the *C. elegans* germline. In rare instances where HR is not used, an error-prone repair pathway termed polymerase theta-mediated end joining (TMEJ) is used to repair germline transposon-induced DSBs (van Schendel et al. 2015).

Transposition can disrupt host–gene function and host–gene regulation (Klein and O’Neill 2018). Due to their high copy number, transposons can also trigger large-scale chromosomal rearrangements (Klein and O’Neill 2018). As a result of these threats, organisms have evolved elaborate defense systems to limit transposon activity. It has been suggested that the regulatory strategies employed against transposons could/should differ in the germline and soma because the evolutionary impacts of transposition differ for both the host and the transposon in these tissues (Haig 2016). For example, successful transposition in the germline is necessary for long-term transposon survival; however, transposition in the soma does not benefit the transposon because it does not lead to a heritable change in copy number (Haig 2016). For host cells, the costs associated with transposition in the germline or soma are typically negative because transposition is more likely to disrupt rather than improve cell/gene function. Additionally, the impacts of germline transposition are likely to be more severe for the host because germline transposition will impact all cells and do so in a heritable manner. In summary, the goal of limiting transposition in the soma is shared by host and transposon, but goals differ in the germline, where transposition is necessary for transposon survival. These considerations predict that the rate of transposon replication should be higher in the germline than in the soma. Consistent with this idea, a germline-specific splicing program ensures that expression of functional *Drosophila P* element transposase is restricted to the germline (Laski et al. 1986; Rio et al. 1986). Thus, it is surprising that studies in the 1980s measuring *Tc1*/*Mariner* class DNA transposon excision rates in the *C. elegans* soma and germline suggest that DNA transposons are ≅1,000-fold more active in the *C. elegans* soma than in the germline (Emmons and Yesner 1984; Eide and Anderson 1985; Eide and Anderson 1988). How or why *Tc1* might be highly active in the *C. elegans* soma is not known.

Here, we show that the non-homologous end joining (NHEJ) machinery is responsible for generating 99.9% of DNA transposon empty sites that occur in post-mitotic, somatic chromosomes during *C. elegans* development. In the absence of NHEJ, there is a near-complete loss of reconstituted empty transposon sites in somatic cell chromosomes. In contrast, loss of NHEJ does not obviously impact transposon biology in the germline, likely because germ cells primarily use homology-based DSB repair pathways to repair transposon-induced DSBs in this tissue (Plasterk 1991; Clejan et al. 2006). Because HR, but not NHEJ, will restore transposons back into their original chromosomal position post-excision, we propose that the apparent ≅1,000-fold elevated somatic activity of DNA transposons in the *C. elegans* soma can, in large part, be explained by differences in DNA repair strategies employed by germ cells and somatic cells to fix their transposon-induced DSBs.

## Materials and methods

### Strains

*C. elegans* strains were cultivated on standard Nematode Growth Medium (NGM) plates at 20°C with *Escherichia coli* strain OP50 as food unless otherwise noted. *glp-1*(*q224ts*) animals were maintained at 15 °C. *Tc1::mScarlet-I* reporter single-copy transgenes were inserted into chromosomal location Chr I: −5.32 cM by CRISPR/Cas9, using a self-excising cassette for selection (Dickinson et al. 2015). SapTrap cloning method (Schwartz and Jorgensen 2016) was used to clone the *Tc1::mScarlet-I* plasmid for CRISPR/Cas9 injection, as previously described (Chang et al. 2025). CRISPR/Cas9 generation of *nhj-1* allele was done by co-CRISPR (Arribere et al. 2014). Because the *nhj-1(vv148)* allele could be present in any strain, we made sure the mutant strains used for assaying *Tc1* excision phenotypes (*rde-3*, *cku-70*, *cku-80*, *lig-4*) did not have the *vv148* allele by PCR genotyping. Strains used are listed in Supplementary Table 1.

### Soma *Tc1* excision quantification

*Tc1::mScarlet-I* reporter quantifications: Individual larval stage 4 (L4) animals were placed onto 6 cm NGM plates, and their progeny was allowed to grow at 20 °C for 4 days. Each plate was then visually scored under the AxioZoom.v16 fluorescence microscope (Zeiss) with the ApoZ 1.5x/0.37 objective for the presence of somatic cell(s) with mScarlet-I fluorescence. The percentage of progeny animals with somatic mScarlet-I fluorescence on a single plate was obtained. For *glp-1* experiments, animals were bleach-synchronized and grown from the embryonic stage at 15 or 25 °C until they reached the L4 stage, then the plates were visually scored as described earlier. All visual scoring of soma fluorescence was done with the examiner blinded to genotype. *Tc1::sfgfp* reporter quantifications: Individual L4 animals were placed onto 6 cm NGM plates, and their progeny was allowed to grow at 20 °C for 3 days. Animals were then washed off the plate with M9 buffer, immobilized with 0.05% sodium azide, and mounted on glass slides. Slides were examined under the Axio Observer.Z1 fluorescent microscope (Zeiss) with the Plan-Apochromat 20x/0.8 M27 objective, and animals were visually scored for the presence of somatic cells expressing sfGFP to determine the percentage of progeny animals on a single plate with somatic sfGFP fluorescence.

### Germline *Tc1* excision rate quantification

Germline *Tc1* excision rate quantifications were done as previously described (Chang et al. 2025).

### Whole genome sequencing and analysis

*C. elegans* genomic DNA was extracted using Gentra Puregene Tissue Kit (Qiagen, #158667), and DNA library preparation was performed using NEBNext Ultra II FS DNA Library Prep with Sample Purification Beads Kit (New England Biolabs, #E6177). Whole-genome sequencing was performed using an Illumina NextSeq instrument, with an Illumina NextSeq Mid-Output 300-cycle kit to obtain paired-end 150 bp reads at 30× coverage (Biopolymers Facility, Harvard Medical School). The quality of the library was assessed using *fastqc* (https://www.bioinformatics.babraham.ac.uk/projects/fastqc/). Paired-end reads for each library were mapped to the reference genome of *C. elegans* (PRJNA13758, WBcel235) using *bwa mem* v0.7.17 (http://bio-bwa.sourceforge.net/bwa.shtml). Compatible SAM files were merged, then sorted into BAM files using *samtools* v1.3.1 (Danecek et al. 2021). The BAM file was indexed to create BAI files using *samtools* to load onto Integrative Genomics Viewer (IGV) (Robinson et al. 2011). The BAM and BAI files were loaded onto IGV, and manual inspection identified a location with unmapped reads in the gene *nhj-1/scb-1* in L strains, but not in the H strain.

### *Tc1* or *Tc3* excision qPCR assay

**Fig. 1.**
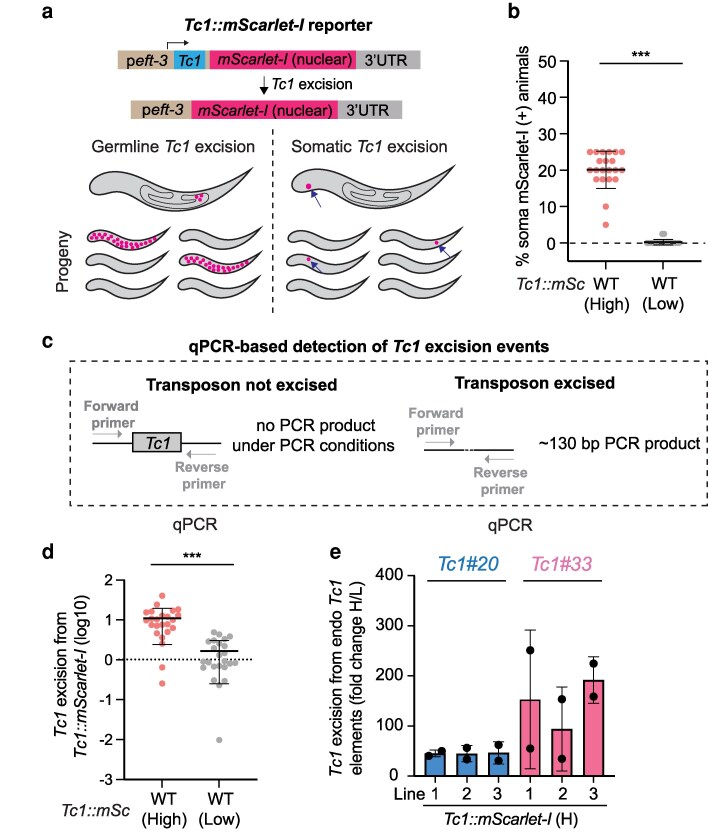
Two ostensibly wild-type strains exhibit different levels of *Tc1* excision in somatic cells. a) (Top) Schematic of *Tc1::mScarlet-I* transposon excision reporter gene used to visualize *Tc1* excision in individual cells. (Lower left) A germline *Tc1* excision event is depicted. In the next generation, progeny inheriting the *Tc1*-excised allele express mScarlet-I in all cells of the animal. (Lower right) A somatic *Tc1* excision event, which results in fluorescent protein expression in one somatic cell, is depicted (arrow). Excision events are not inherited. mScarlet-I expressing cells in progeny (arrows) represent *de novo* excision events. b) *Tc1::mScarlet-I* was inserted into the same chromosomal site in 2 ostensibly wild-type strains of *C. elegans* from the Kennedy lab. Percentage of animals expressing mScarlet-I in one or more somatic cell(s) in these 2 strains is shown. Ostensibly wild-type animals exhibiting high numbers of mScarlet-I (+) cells are termed (High, H). Ostensibly wild-type animals exhibiting low levels of mScarlet-I (+) cells are termed (Low, L). *n* = 21. Error bars are standard deviations (SD). ***, *P* ≤ 0.001(Mann-Whitney test). *Tc1::mSc*, *Tc1::mScarlet-I*. c) Schematic of qPCR assays used to measure *Tc1* excision from *Tc1::mScarlet-I* (d) or endogenous *Tc1* elements (e). The qPCR protocol uses a short elongation time, which restricts productive PCR amplification to shorter, transposon-lacking DNA. Genomic DNA is used as template. d) qPCR assay quantifying *Tc1* excision from *Tc1::mScarlet-I* in (High) or (Low) animals. *Tc1* excision levels were normalized to the mean excision level of *Tc1::mScarlet-I* (Low) animals. *n* = 24 per genotype. Error bars are SD. ***, *P* ≤ 0.001(Mann-Whitney test). e) qPCR quantifying *Tc1* excision from 2 endogenous *Tc1* elements (WBTransposon00000020, *Tc1#20*, and WBTransposon00000033, *Tc1#33*) in 3 independently CRISPR/Cas9-generated *Tc1::mScarlet-I* insertion lines in H animals (lines 1–3) relative to *Tc1* excision in *Tc1::mScarlet-I* (L) animals. *n* = 2. Error bars are SD.

L4 animals were singled onto 6 cm NGM plates and progeny allowed to grow at 20 °C. Four days later, whole-plate worm lysates were obtained by washing animals off the plate and lysing with worm lysis buffer (50 mM KCl, 10 mM pH 8.3 Tris, 2.5 mM MgCl_2_) with proteinase K (0.2 mg/ml). Lysates were used for qPCR assays using the iTaq Universal SYBR Green Supermix (Bio-Rad, 1725121). qPCR assays were run on CFX Connect Real-Time PCR Detection System (Bio-Rad, 1855201) using the following qPCR conditions: (1) Initial denaturation at 95 °C for 5 minutes; (2) 40 cycles of denaturation at 95 °C for 15 seconds and annealing/extension/plate read at 61 °C for 30 seconds. *eft-3* was used as the housekeeping gene. *Tc1* or *Tc3* excision fold changes were calculated using the comparative Cq method (Livak and Schmittgen 2001). If Cq value was undetectable by the end of the 40-cycle qPCR run, Cq value of 41 was used for the fold change calculation. For graphs showing transposon excision fold changes relative to the mean level of excision in a particular genotype, the mean Cq value of that genotype being normalized to was used to calculate relative fold changes. For Figure 1d, for each sample, 5 L4 animals of each genotype were lysed for the qPCR assay instead of using whole plate worm lysates, and lysates were treated with the restriction enzyme PspGI (New England Biolabs) for 1 hour before performing qPCR. *Tc1* sequence harbors 3 PspGI cut sites, and this digestion step was used to prevent *Tc1*-containing genomic templates from amplifying during qPCR. qPCR experiments other than the one in Figure 1d did not use this digestion step, as this step was later found to be not needed. For *glp-1* experiments, animals were bleached, synchronized, and grown from the embryonic stage at 25 or 15 °C. When animals reached larval stage 4, whole plate lysates were obtained and used for qPCR as described earlier. Primers used for qPCR analysis are as follows: *eft-3* F: 5'-GTGAACGTGGTATCACCATC-3'; *eft-3* R: 5'-CGTACCAGTGATCATGTTC-3'; *Tc1*#20 F: 5'-CCGATCATCAATCATAGCG-3'; *Tc1*#20 R: 5'-GAGAACATTTGTGCGAG-3'; *Tc1*#33 F: 5'-GCGAGAAAAGGTATACTC-3'; *Tc1*#33 R: 5'-GTAGACATTATGCACCATTC-3'; *Tc3*#62 F: 5'-CGATTACAGAAGCCATCC-3'; *Tc3*#62 R: 5'-GGTCTCATCAGTAGTGACTC-3'.

## Results

### Ostensibly wild-type strains of *C. elegans* exhibit radically different levels of apparent transposon activity in the soma

*C. elegans Tc1* is a *Tc1/Mariner* class DNA transposon which is active in the *C. elegans* soma and germline (Plasterk 1996). To enable exploration of the biology of transposons in an intact animal system, we engineered reporter genes (*Tc1::mScarlet-I* and *Tc1::sfgfp*) that allow visualization of *Tc1* excision events at the single-cell level in the *C. elegans* soma and germline (Chang et al. 2025). In these reporters, *Tc1* excision in the germline results in expression of mScarlet-I or sfGFP fluorescent proteins in all cells of the progeny animal, and this expression state is heritable (Fig. 1a) (Chang et al. 2025). *Tc1* excision in the soma results in animals expressing fluorescent proteins in 1 or more somatic cells, and this expression pattern is not inherited (Fig. 1a) (Chang et al. 2025). In the *Tc1::mScarlet-I* reporter gene, *Tc1* is inserted into the promoter of *Tc1::mScarlet-I*, and mScarlet-I is expressed when *Tc1* excises from the reporter and leaves behind no scar or small insertions/deletions (indels) (Fig. 1a) (Chang et al. 2025). In the *Tc1::sfgfp* reporter gene, the reading frame of *sfgfp* is restored only when *Tc1* excision results in a + 4 insertion scar, which is the typical (78%) footprint left after *Tc1* excision in the soma (Eide and Anderson 1988). Using the *Tc1::mScarlet-I* reporter, we previously quantified *Tc1* excision rates in the germline and soma of *C. elegans* and observed a ≅1,000-fold higher *Tc1* excision rate in somatic cells than in germ cells (Chang et al. 2025). This data is consistent with previous reports, which assessed *Tc1* excision by Southern blotting or by phenotypic restoration of UNC-54 function following *Tc1* excision from *unc-54::Tc1* alleles (Emmons and Yesner 1984; Eide and Anderson 1985; Eide and Anderson 1988). Historically, the terms transposon excision and transposon empty site formation have been used interchangeably with regards to transposon mobilization, and indicate the end point of a transposon mobilization event (Supplementary Fig. 1). Transposon excision, as measured by Southern blotting or genetic reporters such as *unc-54::Tc1*, *Tc1::mScarlet-I* and *Tc1::sfgfp* necessitates that host chromosomes, from which a transposon has excised, are repaired by an error-prone host DNA repair system that reconnects 5' and 3' termini following transposon excision. Henceforth, we use the phrase “transposon excision” to refer to a chromosomal site from which a DNA transposon has excised and for which the host DNA repair machinery regenerated an intact chromosome, lacking a transposon (Supplementary Fig. 1). Note that this definition does not include excision events in which transposon-induced DSBs have been created and are repaired via a non-error prone DSB repair pathway.

While using our transposon reporter genes to explore how and why *Tc1* might be highly active in the soma, we were surprised to find that introduction of *Tc1::mScarlet-I* into the identical chromosomal position in 2 ostensibly isogenic N2 Bristol (wild-type) strains of *C. elegans* resulted in animals exhibiting radically different numbers of fluorescent cells in the soma. More specifically, when we used CRISPR/Cas9 to insert a *Tc1::mScarlet-I* reporter gene into the same chromosomal site in 2 N2 (wild-type) strains from the Kennedy lab, we observed 2 radically different results: In 1 ostensibly wild-type strain, 20% of animals expressed high levels of mScarlet-I in 1 or more somatic cells, while in another ostensibly wild-type strain only 0.2% of animals expressed mScarlet-I in the soma (Fig. 1b). We did not observe an obvious pattern to the types of somatic cells that expressed mScarlet-I in the 2 ostensibly wild-type strains, hinting that *Tc1* excision occurs in many/most somatic cell types in both strains. Henceforth, we refer to ostensibly wild-type strains that exhibit low numbers of somatic fluorescent cells as *Tc1::mScarlet-I* (Low, L) and ostensibly wild-type strains with high numbers of somatic fluorescent cells as *Tc1::mScarlet-I* (High, H). The data described earlier suggest that H and L animals might accumulate radically different numbers of empty *Tc1* sites in somatic chromosomes. We used qPCR-based assays to test this idea (Fig. 1c). Indeed, qPCR-based assays showed that the number of empty *Tc1::mScarlet-I* transposon sites in H animals was about 10-fold higher than in L animals (Fig. 1d). To assess if this difference in H and L animals was limited to the *Tc1::mScarlet-I* reporter gene, or applied more generally to endogenous *Tc1* elements, we used qPCR to quantify *Tc1* excision from 2 endogenous *Tc1* elements. Similar to what was observed for the *Tc1::mScarlet-I* reporter gene, 3 independently generated lines of *Tc1::mScarlet-I* (H) animals showed ≅40 to 200-fold elevated levels of excision from endogenous *Tc1* elements when compared to L animals (Fig. 1e). Taken together, the data support the idea that H animals exhibit higher rates of *Tc1* excision than L animals.

### H/L segregate as a Mendelian trait

**Fig. 2.**
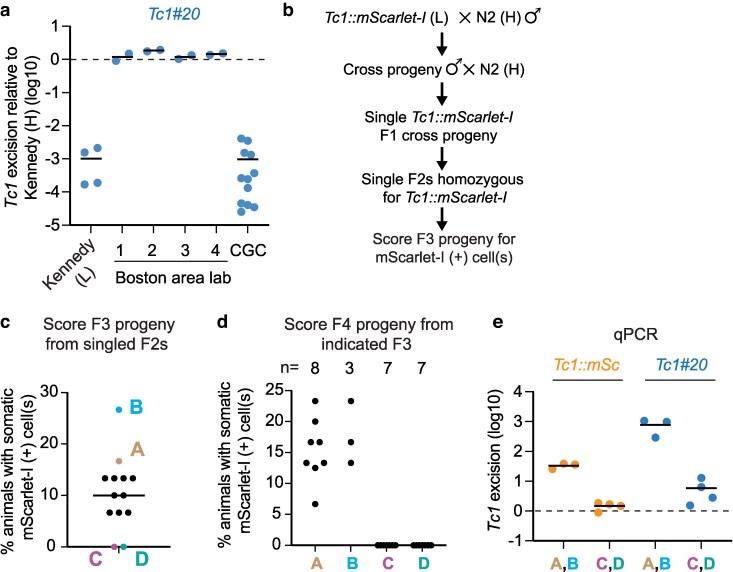
H/L phenotypes segregate as a Mendelian trait. a) qPCR assay for *Tc1* excision from an endogenous *Tc1* element (*Tc1#20*) in the Kennedy lab (L) strain, wild-type strains obtained from 4 different labs in the Boston area (Boston area lab 1–4, see Supplementary Table 1 for strain list), and the CGC. *Tc1* excision rates are shown relative to the Kennedy lab (H) strain. b) Outline of genetic cross between WT (H) and *Tc1::mScarlet-I* (L) animals. *Tc1::mScarlet-I* (L) was crossed to WT (H) twice because this cross was originally intended to serve as a control for other crosses, which are not described further here. c) Quantification of % of F3 progeny- from F2's as outlined in (b)- that expressed mScarlet-I in 1 or more somatic cells. Bar shows median value. (d,e) Further analysis of select F2 lineages exhibiting a High, H phenotype (lines A, B) or a Low, L (lines C, D) phenotype. d) % of F4 progeny- from indicated F3's- that expressed mScarlet-I in 1 or more somatic cells is shown. n indicates the number of F4 populations, each from different F3s, that were scored. e) *Tc1* excision from *Tc1::mScarlet-I* and *Tc1#20* quantified by qPCR assays in lines established from F2 lineages A and B (A, B) or C and D (C, D) are shown. Excision levels are plotted as fold changes relative to that of *Tc1::mScarlet-I* (L) animals. Bars show mean values.

One explanation for the results outlined above is that one of the 2 Kennedy lab ostensibly wild-type strains carries a genetic modifier that dramatically impacts *Tc1* excision in the *C. elegans* soma. To begin testing this idea, we assayed 5 additional wild-type strains that were obtained from 4 other *C. elegans* labs or from the *Caenorhabditis* Genetics Center (CGC) strain depository for *Tc1* excision rates from an endogenous *Tc1* element. The analysis showed that 4 of these strains exhibited the H phenotype and 1 (CGC) exhibited the L phenotype (Fig. 2a), consistent with the idea that a polymorphic allele, which affects *Tc1* excision in the soma, is present in a subset of ostensibly wild-type *C. elegans* strains. To test this idea further, we conducted a genetic cross between H and L strains (Fig. 2b). We singled F2 progeny and scored mScarlet-I expression in the F3 progeny of these F2 animals. The analysis identified 2 F2 lineages exhibiting the H phenotype, 2 lineages exhibiting the L phenotype, and 9 lineages exhibiting intermediate phenotypes (Fig. 2c). In the next generation, the H/L phenotypes of H/L lineages bred true (Fig. 2d). Similar results were obtained from an independent cross between H and L animals (Supplementary Fig. 2). qPCR-based analyses demonstrated that the H and L phenotypes, as defined by rates of mScarlet-I expression in somatic cells, correlated with levels of *Tc1* excision from *Tc1::mScarlet-I* and from an endogenous *Tc1* element as assessed by qPCR (Fig. 2e). Thus, H/L segregates as a Mendelian trait, suggesting that the rate at which somatic cells undergo *Tc1* excision is under genetic control and that a polymorphic allele is present in some ostensibly wild-type strains of *C. elegans* that dramatically alters the rate of *Tc1* excision in the soma.

### NHJ-1 prevents *Tc1* excision events in the soma

**Fig. 3.**
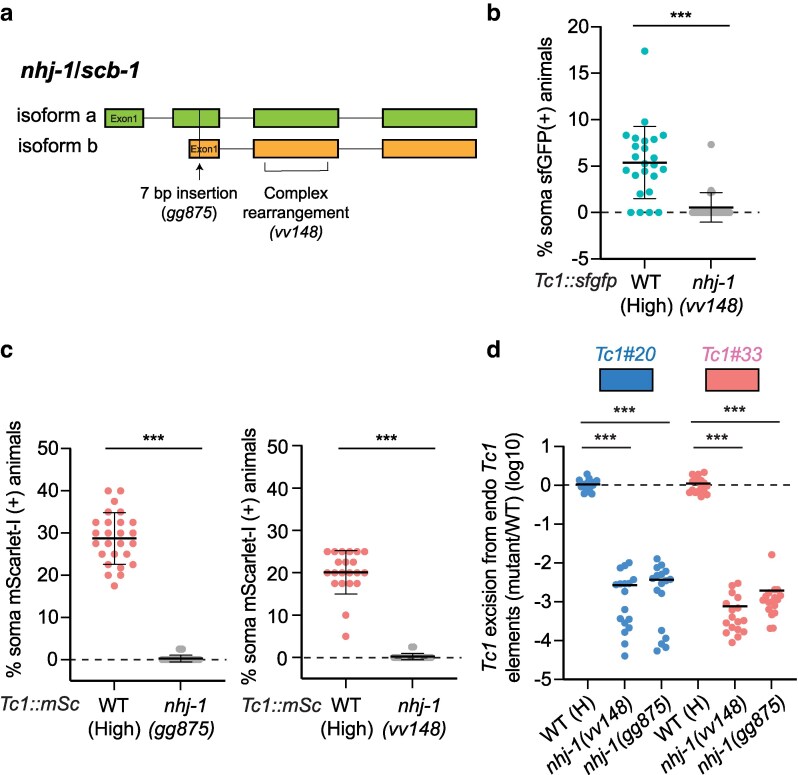
*nhj-1* mutation is causative for suppression of *Tc1* excision. a) Schematic of putative *nhj-1/scb-1* isoforms a and b gene structures. Not to scale. Colored block, exon; line, intron. Alleles *gg875* (a 7 base pair “TAATCTA” insertion) and the complex rearrangement *vv148* are indicated. b) Percentage of *Tc1::sfgfp* animals of indicated genotypes expressing sfGFP in 1 or more somatic cells is shown. *n* = 25 per genotype. Error bars are SD. ***, *P* ≤ 0.001 (Mann-Whitney test). c) Percentage of *Tc1::mScarlet-I* animals of indicated genotypes expressing mScarlet-I in 1 or more somatic cells is shown. *n* = 21–27 per genotype. Error bars are SD. ***, *P* ≤ 0.001 (Mann–Whitney test). Graph on the right is the same as Figure 1b. d) qPCR-based quantification of *Tc1* excision from *Tc1#20* and *Tc1#33*. Data are represented as log10 fold change relative to the mean level of transposon excision in (H) animals. Bars show mean values. ***, *P* ≤ 0.001 (Kruskal–Wallis test with Dunn's test for multiple comparisons).

We sought to identify the gene(s) responsible for regulating rates at which empty *Tc1* sites accrue in the soma. Data presented subsequently will show that the gene responsible for H/L phenotypes is *nhj-1*/*scb-1*, and that the mutant allele is present in L animals. For clarity, we refer to this gene henceforth as *nhj-1.* To positionally map *nhj-1*, we crossed L animals to a strain from the Million Mutation Project (Thompson et al. 2013), VC40641, which exhibited an H phenotype (Supplementary Fig. 3a). We isolated 75 recombinant F2 progeny exhibiting the L phenotype and used sequence polymorphisms present in the 2 parent strains to map *nhj-1* to Chr V, between 9.4 and 12.6 Mb (Supplementary Fig. 3b). We then conducted whole-genome sequencing of L and H animals. Surprisingly, variant calling programs (samtools/bcftools in the Mutation Identification in Model Organism Genomes (MiModD) software package (Maier 2018)) failed to identify any DNA alterations between H and L animals within our mapping interval. However, manual inspection of the sequencing data identified a small region within the gene *nhj-1*, located at 11.1 Mb on Chr V, in which sequencing coverage was ≅20-fold higher in H animals than L animals (Supplementary Fig. 4). A recent study identified a small deletion, followed by a repetitive insertion, within the *nhj-1* gene (termed *nhj-1(vv148)*), which is present in a subset of ostensibly wild-type strains of *C. elegans* (Fig. 3a) (Vujin et al. 2020). *nhj-1* encodes a protein needed for *C. elegans* to repair DSBs induced by chemotherapeutic agents and ionizing radiation (Brady et al. 2019; Evans and Andersen 2020; Vujin et al. 2020). PCR-based genotyping analyses showed that *vv148* was present in our L animals, but not H animals (Supplementary Fig. 5). Three additional results establish that *nhj-1(vv148)* is causative for lowering *Tc1* excision levels. First, we introduced *nhj-1(vv148)* into animals expressing the *Tc1::sfgfp* reporter gene by genetic cross and observed that *nhj-1(vv148)* animals exhibited a lowered rate of *Tc1* excision, as measured by somatic sfGFP expression (Fig. 3b). Second, we used CRISPR/Cas9 to generate an independent *nhj-1* allele in *Tc1::mScarlet-I* (H) animals. The resultant allele (termed *gg875*), a 7-base-pair insertion of the sequence TAATCTA, is predicted to be a strong loss-of-function for *nhj-1* as it introduces a premature stop codon, and puts the majority of *nhj-1* out of frame (Fig. 3a). *nhj-1(gg875); Tc1::mScarlet-I* animals exhibited reduced *Tc1* excision in the soma, as assessed by counting mScarlet-I expressing somatic cells in these animals (Fig. 3c). *gg875* lowered somatic *Tc1* excision to a similar degree as *nhj-1(vv148)* (Fig. 3c). Finally, qPCR-based assays showed a similar decrease in *Tc1* excision levels from endogenous *Tc1* elements in *gg875* and *vv148* animals (Fig. 3d). The data establish that loss of NHJ-1 radically lowers the number of *Tc1* empty sites that accrue in the soma of *C. elegans*.

### NHJ-1 acts in the soma, but not the germline, to limit *Tc1* excision

**Fig. 4.**
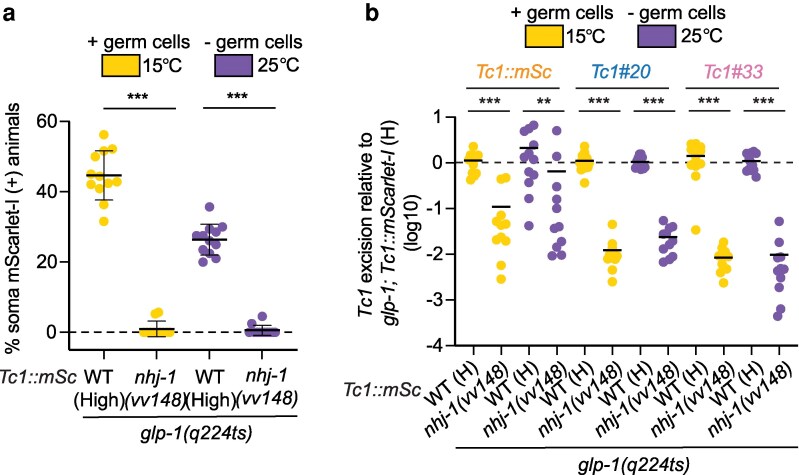
*nhj-1* mutation acts independently of the germline to suppress *Tc1* excision. a) Percentage of animals of the indicated genotypes expressing mScarlet-I in 1 or more somatic cell(s) is shown. Animals are *glp-1(q224ts); Tc1::mScarlet-I*, with or without *nhj-1(vv148)*. Animals were grown at the permissive temperature for *glp-1(q224ts)* 15 °C (animals produce germ cells, + germ cells), or the non-permissive temperature 25 °C (animals lack 99% of germ cells,—germ cells). *n* = 11–12. Error bars are SD. ***, *P* ≤ 0.001 (Mann–Whitney test). b) qPCR-based quantification of *Tc1* excision from *Tc1::mScarlet-I, Tc1#20*, or *Tc1#33* in animals with genotypes- and grown at temperatures- outlined in (a). Data is log10 and is relative to mean level of *Tc1* excision in *glp-1(q224ts); Tc1::mScarlet-I* animals at the same temperature. *n* = 9–12. Bars show mean values. **, *P* ≤ 0.01; ***, *P* ≤ 0.001 (Kruska–Wallis test with Dunn's test for multiple comparisons).

To ask if NHJ-1 functions in the soma to regulate *Tc1* excision, and not indirectly via the germline, we asked if *nhj-1(−)* animals lacking germ cells still exhibited reduced *Tc1* excision. Indeed, *glp-1(q224ts)* animals, which lack 99% of germ cells when grown at the non-permissive temperature of ≥25 °C (Beanan and Strome 1992), still exhibited suppressed *Tc1* excision in an *nhj-1(−)* background, comparable to *nhj-1(−)* animals with intact germlines (Fig. 4a). Similar results were observed when we used qPCR to assay *Tc1* excision from endogenous *Tc1* elements (Fig. 4b). The data suggest that NHJ-1 regulates *Tc1* excision in the soma. We next asked directly if NHJ-1 regulates *Tc1* excision in the germline. A previous study demonstrates that germline excision of *Tc1* from *Tc1::mScarlet-I* can be assessed using the *Tc1::mScarlet-I* reporter gene (Chang et al. 2025). To assess if NHJ-1 regulates *Tc1* excision in the germline, we grew *nhj-1(+); Tc1::mScarlet-I* and *nhj-1(−); Tc1::mScarlet-I* animals to high density over several generations and then determined the percentage of growth plates containing 1 or more animals expressing mScarlet-I in all cells of the soma and germline, which is indicative of at least 1 germline *Tc1* excision event. A Poisson distribution method (see Materials and Methods) was then used to set a maximum *Tc1* excision rate (Collins et al. 1987; Eide and Anderson 1988). The analysis showed that loss of NHJ-1 did not obviously affect the rate of *Tc1* excision in the germline, at least to levels detectable by this assay (Table 1). To address detection limits of the assay, we asked whether *nhj-1(−)* might affect germline *Tc1* excision rates in *rde-3(−)* animals, where *Tc1* excision is ≥10^4^ times more frequent than in wild-type animals (Chang et al. 2025). We did not observe a significant change in germline *Tc1* excision in *rde-3(−); nhj-1(−)* animals when compared to *rde-3(−)* animals (Table 1). The results are consistent with previous reports showing that TMEJ, not NHEJ, is the primary error-prone repair pathway for repair of transposon-induced DSBs in *C. elegans* germ cells (Robert and Bessereau 2007; van Schendel et al. 2015). We conclude that 1 function of NHJ-1 is to regulate *Tc1* excision, and that this regulation occurs predominantly in the soma.

**Table 1.** Germline *Tc1* excision rates determined using the *Tc1::mScarlet-I* reporter.

Genotype	# plates scored	# animals per plate (F1)	# animals per plate (F2/3)	# plates with animal(s) expressing mScarlet-I in all cells	Germline *Tc1* excision rate
Wild-type	167	ND	29,120	0/122	<2.8 × 10^−7^
*nhj-1(gg875)*	39	ND	26,309	0/39	<9.9 × 10^−7^
*rde-3(ne3370)*	81	322	ND	54/81	3.4 × 10^−3^
*rde-3(ne3370); nhj-1(vv148)*	34	435	ND	26/34	3.3 × 10^−3^
*rde-3(ne3370); lig-4(ok716)*	28	240	ND	18/28	4.2 × 10^−3^

Animals of indicated genotypes (P0) were allowed to grow until F1 or F2/3 generations (as indicated), then scored for the presence of ≥1 animal(s) heritably expressing mScarlet-I in all cells (indicative of germline *Tc1* excision event(s)). Genotypes with low germline *Tc1* excision rates were scored at F2/F3 generation (wild-type, *nhj-1*). Genotypes with high germline *Tc1* excision rates were scored at F1 generation (all *rde-3(−)* allele-containing animals). Germline *Tc1* excision rates (f) were calculated using the Poisson distribution formula: f = − (ln(*N*/*T*))/*n* (Collins et al. 1987; Eide and Anderson 1988). *T*: total number of plates scored, *N*: total number of plates without any germline *Tc1* excision event, *n*: average number of animals per plate. See Materials and Methods for additional information regarding methods. Data are aggregates from scoring at least 2 independent experiments per genotype. ND, not determined.

### NHJ-1 is a general enhancer of *Tc1*/*Mariner* class DNA transposon excision

We asked if NHJ-1 was a general enhancer of *Tc1*/*Mariner* class DNA transposon excision by measuring excision rates for the *Tc3* transposon, which lacks significant sequence homology with *Tc1* (Collins et al. 1989). The analysis revealed a 10-fold lower *Tc3* excision rate in *nhj-1(−)* animals compared to *nhj-1(+)* animals (Supplementary Fig. 6). The reason that *Tc3* excision rates decrease 10-fold- while *Tc1* rates decrease 100 to 1,000-fold—in *nhj-1* mutants is not yet known (compare Supplementary Fig. 6 and Fig. 3d) but may relate to differing PCR efficiencies in these qPCR assays. The data suggest that NHJ-1 is a general promoter of *Tc1*/*Mariner* class DNA transposon excision.

### NHEJ is needed for *Tc1* excision in somatic cells

**Fig. 5.**
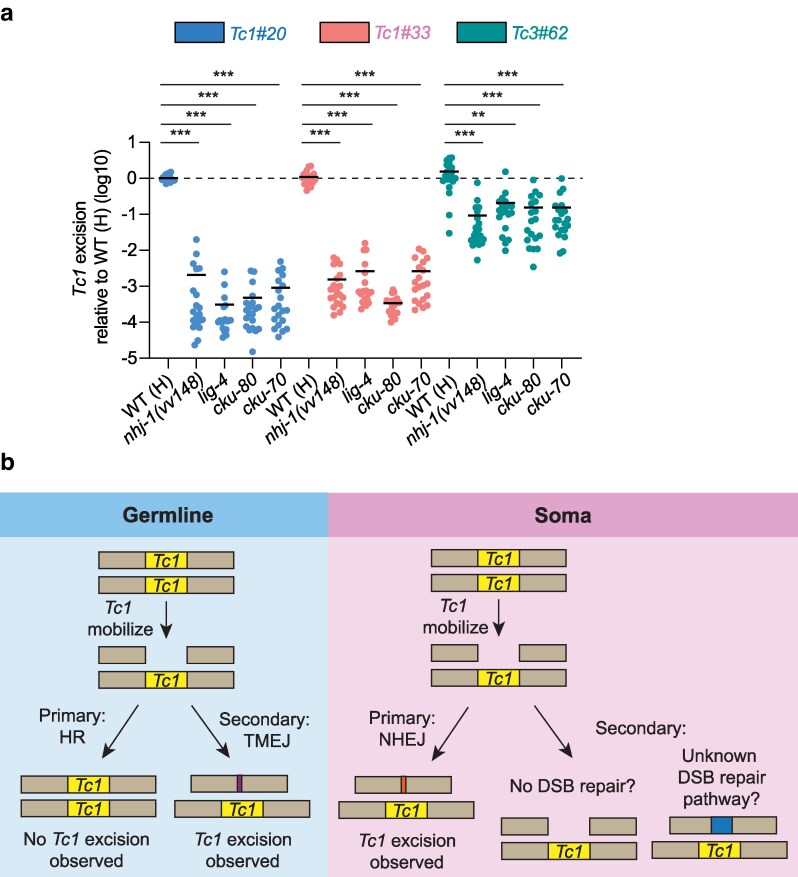
NHEJ pathway is needed for *Tc1*/*Mariner* class transposon excision in the soma. a) qPCR assay quantifying transposon excision from *Tc1#20*, *Tc1#33*, and *Tc3#62* in animals of the indicated genotypes. Alleles: *nhj-1(vv148)*, *lig-4(ok716)*, *cku-80(ok861)*, *cku-70(tm1524)*. Data are log10 fold and are shown relative to the mean level of transposon excision in WT (H) animals. *n* = 19–20. Bars show mean values. **, *P* ≤ 0.01; ***, *P* ≤ 0.001 (Kruskal–Wallis test with Dunn's test for multiple comparisons). *Tc3#62*, WBTransposon00000062. b) Model showing how DSB repair pathway usage can account for the high rates of *Tc1* excision in the *C. elegans* soma relative to the germline. (Left) DSBs formed by *Tc1* mobilization in the germline are primarily repaired by HR, restoring *Tc1* elements back into their chromosomal positions. Only in rare instances, TMEJ is used. (Right) DSBs formed by *Tc1* mobilization in the soma are primarily repaired by NHEJ, which creates empty *Tc1* sites. In the absence of NHEJ, DSBs formed by *Tc1* mobilization in the soma are either not repaired or are repaired by an unknown DSB repair pathway, and results in *Tc1* excision events being rarely observed. Thus, by altering the DSB repair of somatic cells, *Tc1* excision in the soma becomes not apparent, similar to the germline. Note that we have not used our reporter system to test the extent to which alteration of DSB repair by loss of HR can elevate *Tc1* excision rates in the germline. DSB, double-strand break. HR, homologous recombination. TMEJ, polymerase theta-mediated end joining. NHEJ, non-homologous end joining.

The NHEJ pathway repairs DSBs by directly ligating together broken DNA ends. Three core components of the eukaryotic NHEJ machinery, LIG-4, CKU-80, and CKU-70, are conserved in *C. elegans* (Clejan et al. 2006; Chang et al. 2017). A previous study finds that *C. elegans nhj-1* acts in a genetic pathway with *lig-4*, *cku-80*, and *cku-70* to repair DSBs (Vujin et al. 2020). Therefore, the ability of NHJ-1 to promote *Tc1* excision in the *C. elegans* soma could relate to its role in NHEJ-based DSB repair. To test this idea, we asked if animals harboring loss-of-function mutations in *lig-4*, *cku-80*, or *cku-70* also exhibited lowered rates of *Tc1* excision. We used qPCR to monitor *Tc1* excision at 2 endogenous *Tc1* elements and 1 endogenous *Tc3* element in wild-type or *lig-4*, *cku-80*, *cku-70* mutants. We observed that *lig-4*, *cku-80*, and *cku-70* mutations caused *Tc1* excision levels to decrease by 100 to 1,000-fold and *Tc3* excision levels to decrease by 10-fold (Fig. 5a). Note that the *lig-4*, *cku-80*, and *cku-70* animals used in this study were genotyped to ensure they did not harbor *nhj-1(vv148)*. The results show that loss of any component of the NHEJ DSB repair machinery leads to a dramatic decrease in *Tc1* excision in *C. elegans*. Similar to what we observed for *nhj-1(−); rde-3(−)* animals, *lig-4* mutation did not suppress germline *Tc1* excision in *rde-3(−)* animals (Table 1). The simplest interpretation of this data is that NHEJ is responsible for generating 90%–99.9% of the empty DNA transposon sites present in somatic cells of *C. elegans*.

## Discussion

Here, we show that *C. elegans* lacking the NHEJ DSB repair machinery show a dramatic decrease in the number of empty transposon sites that accumulate in somatic tissues. Our data suggest that during normal growth and development, the repair of transposon-induced DSBs by NHEJ, which ligates broken DNA ends, is almost entirely responsible for generating empty transposon sites that accumulate in the soma (Chang et al. 2017). By contrast, transposon-induced DSBs are preferentially repaired by HR in the *C. elegans* germline, which, by using *Tc1*-containing homologs as templates for repair, masks *Tc1* excision by copying excised transposons back into their original chromosomal positions post-excision (Plasterk 1991). It has been suggested that *Tc1*/*Mariner* class DNA transposons are 1,000-fold more active in the *C. elegans* soma than in the germline (Emmons and Yesner 1984; Eide and Anderson 1985; Eide and Anderson 1988). We propose that this apparent 1,000-fold elevated DNA transposon excision rate in the *C. elegans* soma can be explained largely, if not entirely, by tissue-specific differences in DNA repair strategy, which result in the accumulation of dramatically different numbers of empty transposon sites in the soma and germline (Fig. 5b). It is important to note that while this work provides evidence that DNA repair choice dramatically impacts the apparent rate of transposon excision in *C. elegans*, we do not quantify actual transposon excision rates here. Thus, the precise rates of transposon excision in the *C. elegans* soma and germline are not yet known. Determining these rates will require disabling all DSB repair systems prior to quantifying transposition.

Homology-based repair and NHEJ-based repair pathways are used to repair *P* element transposon-induced DSBs in the germline and soma of flies, respectively (Engels et al. 1990; Gloor et al. 2000), and NHEJ repairs *Tc1*/*Mariner*-like *Sleeping Beauty* transposon-induced DSBs in mammalian somatic cells (Yant Stephen and Kay Mark 2003; Izsvák et al. 2004). Thus, tissue-specific differences in DNA repair strategies could influence apparent transposon excision rates in other animals, and, therefore, DNA repair should be considered when attempting to quantify DNA transposon activity in any organism.

We do not yet know what happens to chromosomes broken by transposon excision in *C. elegans* somatic cells which lack NHEJ. A homology-based repair pathway is used to repair *Sleeping Beauty*-induced DSBs in NHEJ-deficient mammalian cells (Izsvák et al. 2004). It is possible that NHEJ-defective *C. elegans* employ a homology-based repair pathway to fix transposon-induced DSBs, which masks transposon excision events (Fig. 5b). Alternatively, somatic transposon-induced DSBs may never be repaired (Fig. 5b). Indeed, following ionizing radiation exposure, HR does not effectively compensate for loss of NHEJ in somatic cells of *C. elegans* (Clejan et al. 2006). For example, following radiation exposure, NHEJ-defective, but HR-competent, late-stage *C. elegans* embryos exhibit defects in chromosome segregation, which suggests many DSBs are not repaired (Clejan et al. 2006). Our failure to detect fluorescent cells in the soma of NHEJ-defective animals harboring transposon reporter genes is consistent with the idea that transposon-induced DSBs are not repaired. Repairing DSBs is essential in mitotically dividing cells in order to prevent mitotic catastrophes (Castedo et al. 2004). Many *C. elegans* somatic cells, however, cease dividing after embryogenesis (Sulston and Horvitz 1977). Indeed, in *Tc1::mScarlet-I* animals, 71% of animals, which harbored fluorescent cells, possessed a single mScarlet-I positive cell, suggesting that most *Tc1* empty sites, which accrue in the soma, are generated in post-mitotic cells (Supplementary Fig. 7). It is possible that the paucity of mitotically dividing cells in post-embryogenic *C. elegans* makes expressing redundant DSB repair pathways unnecessary for this organism, and therefore, transposon-induced DSBs are never repaired in NHEJ-defective *C. elegans* somatic cells. It will be of interest to assess whether or not post-mitotic somatic *C. elegans* cells repair transposon-induced DSBs, or other DSBs, in the absence of NHEJ. Assays that quantify numbers of DNA termini in NHEJ(+) and NHEJ(-) animals, such as TUNEL assays (Gavrieli et al. 1992), may be able to address this question. If DSBs are not repaired, it will be of interest to explore the biological consequences, if any, of failing to repair broken chromosomes in post-mitotic cells.

We find that in wild-type *C. elegans* NHEJ repairs most transposon-induced DSBs in post-mitotic somatic cells. Previous studies have documented physical and/or genetic interactions between components of the NHEJ machinery and DNA transposon-encoded proteins. For instance, the mammalian NHEJ factor Ku70 interacts physically with the transposase encoded by the *Sleeping Beauty* transposon (Izsvák et al. 2004). Mammalian RAG proteins, which are widely considered domesticated transposases, interact with NHEJ factors (Ku70 and Ku80) to drive V(D)J recombination (Raval et al. 2008). In *Paramecium*, NHEJ and the transposase-like PiggyMac cooperatively mediate programmed DNA elimination in developing macronuclei (Dubois et al. 2012). These interactions hint that the NHEJ machinery and transposases may have co-evolved to interact, perhaps to ensure efficient repair of somatic chromosomes broken by transposon excision events. Assessing if the *C. elegans* NHEJ machinery interacts with transposon-encoded transposases will be a good test of this latter idea.

## Supplementary Material

jkag054_Supplementary_Data

## Data Availability

Strains generated in this study are listed in Supplementary Table 1 and available upon request. Supplemental material available at G3 online.

## Statistical analysis

Statistical analysis was done using GraphPad Prism (www.graphpad.com).
