# Supplementary material for: Tissue-specific DNA repair strategies underlie apparent high transposon activity in the Caenorhabditis elegans soma
Source: G3 (Bethesda). 2026 Mar 18;16(5):jkag054. doi: 10.1093/g3journal/jkag054 (PMC13148408; doi:10.1093/g3journal/jkag054)
Supplement: jkag054_Supplementary_Data [file jkag054_supplementary_data.pdf]

Supplementary Material

Figures S1-S7.

Supplementary Table 1: List of strains

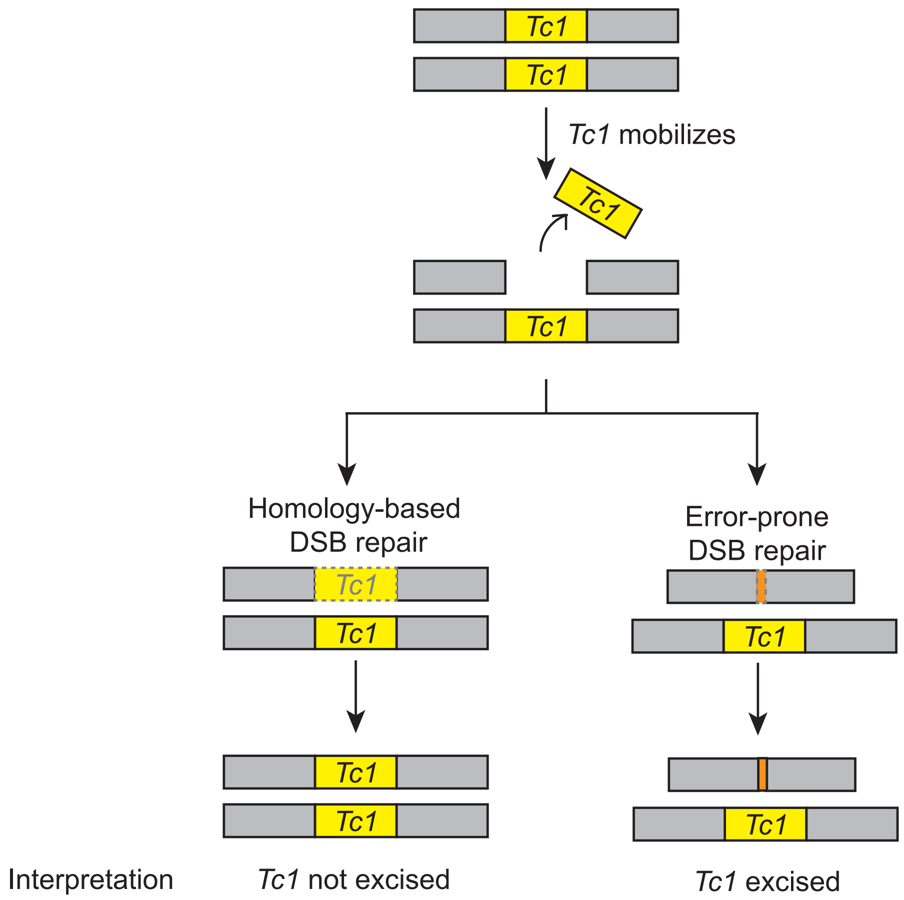

Figure S1. Schematic for definition of *Tc1* excision. Following a transposon mobilization event, double-strand breaks (DSBs) can be fixed via homology-based or error-prone DSB repair pathways. When a homology-based DSB repair pathway is used, the transposon is copied back into its original site, resulting in no loss of *Tc1* from the original mobilization site ("*Tc1* not excised"). When an error-prone DSB repair pathway is used, the transposon excision event is fixed such that the transposon is missing after repair ("*Tc1* excised").

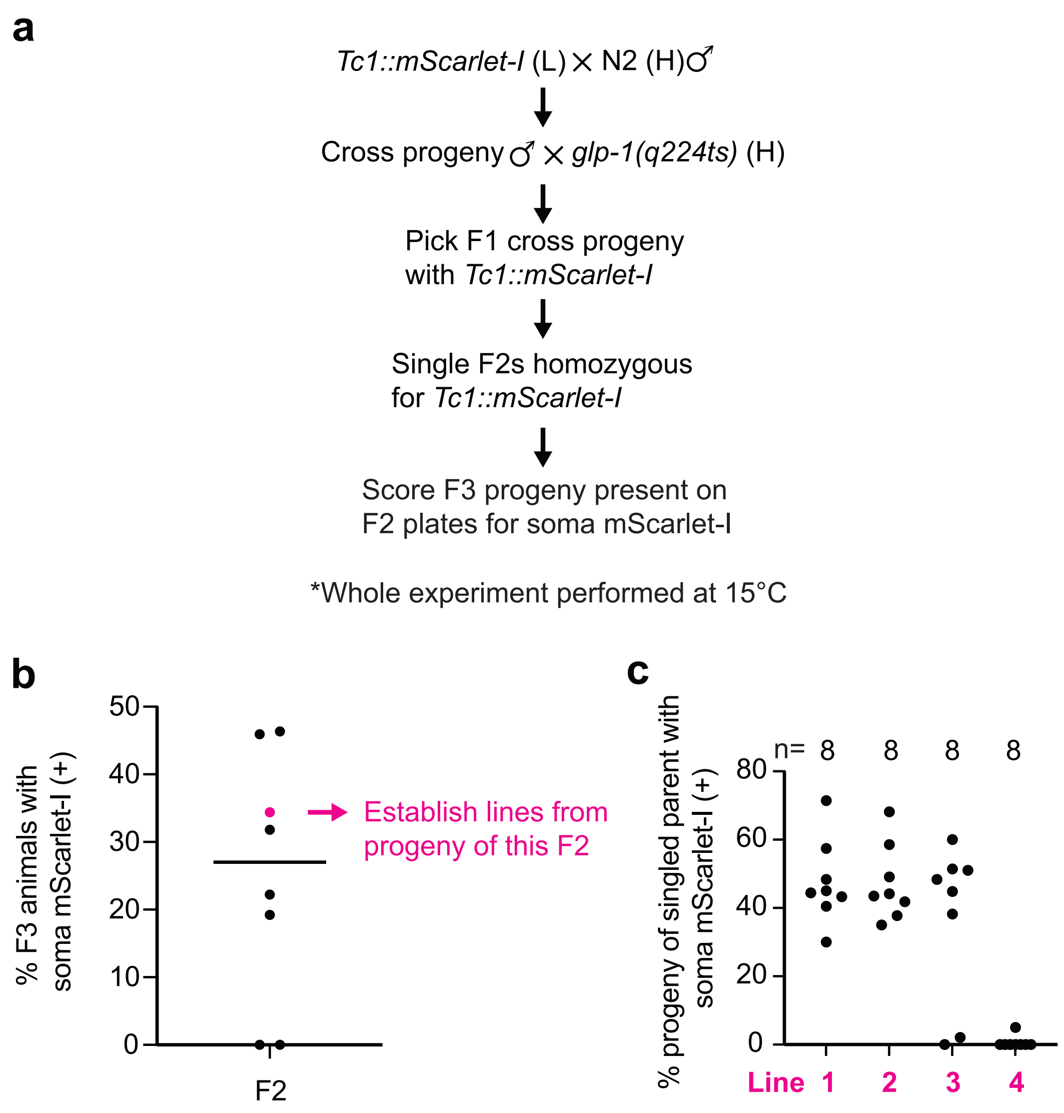

Figure S2. H/L phenotypes segregate as a Mendelian trait. (Related to Figure 2). (a) Schematic of a genetic cross between WT (H) and *Tc1::mScarlet-I* (L). Note, *glp-1(q224ts)* was introduced in the second step of the crossing scheme because this cross was originally designed to investigate the effect of crossing *Tc1::mScarlet-I* (L) to *glp-1(q224ts)*. Cross was performed at 15°C. **(b)** Singled F2 animals from cross outlined in **(a)** were scored for the percentage of F3 progeny expressing mScarlet-I in one or more cells. Bar shows median value. The F2 lineage highlighted in pink was chosen for further evaluation in the next generation **(c)**. **(c)** Four lines (Line 1-4) were established by singling animals from the F2 lineage indicated in pink in **(b)**. Progeny of eight singled animals were scored for percentage of animals expressing mScarlet-I in one or more cells. n indicates the number of populations scored.

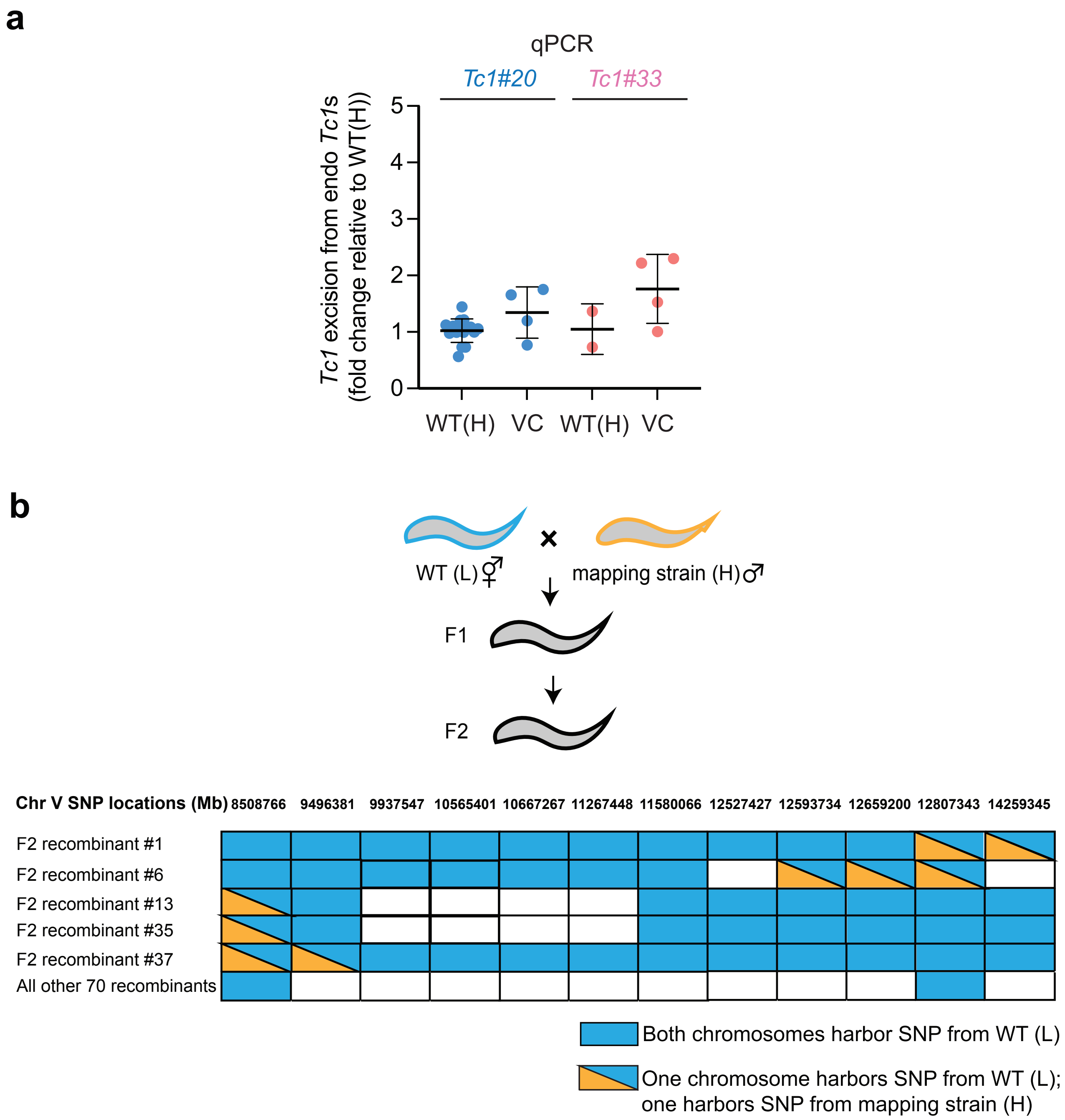

Figure S3. Positional mapping identifies that the causal allele for low soma *Tc1* excision is located on Chromosome V between 9.4 and 12.6 Mb. (a) qPCR assays measuring levels of *Tc1* excision from *Tc1#20* and *Tc1#33*. Fold changes normalized to mean WT (H) excision levels. Error bars show standard deviation (SD). VC, VC40641 strain. **(b)** (Top) Genetic cross between WT (L) and mapping strain (Million Mutation Project strain (VC40641)) (H) for positional mapping. (Bottom) Seventy-five F2 progeny isolated from the positional mapping cross (top) with L phenotypes were genotyped for single nucleotide polymorphisms (SNPs) in VC40641. Five informative F2 recombinants are shown; the remaining 70 F2 animals were homozygous for SNP markers of WT (L) at the Chr V 9.4-12.6 Mb region. Blue bar: Both chromosomes harbor SNP from WT (L). Blue/orange bar:

One chromosome harbors WT (L) SNP, the other chromosome harbors VC40641 SNP. SNP, single nucleotide polymorphism.

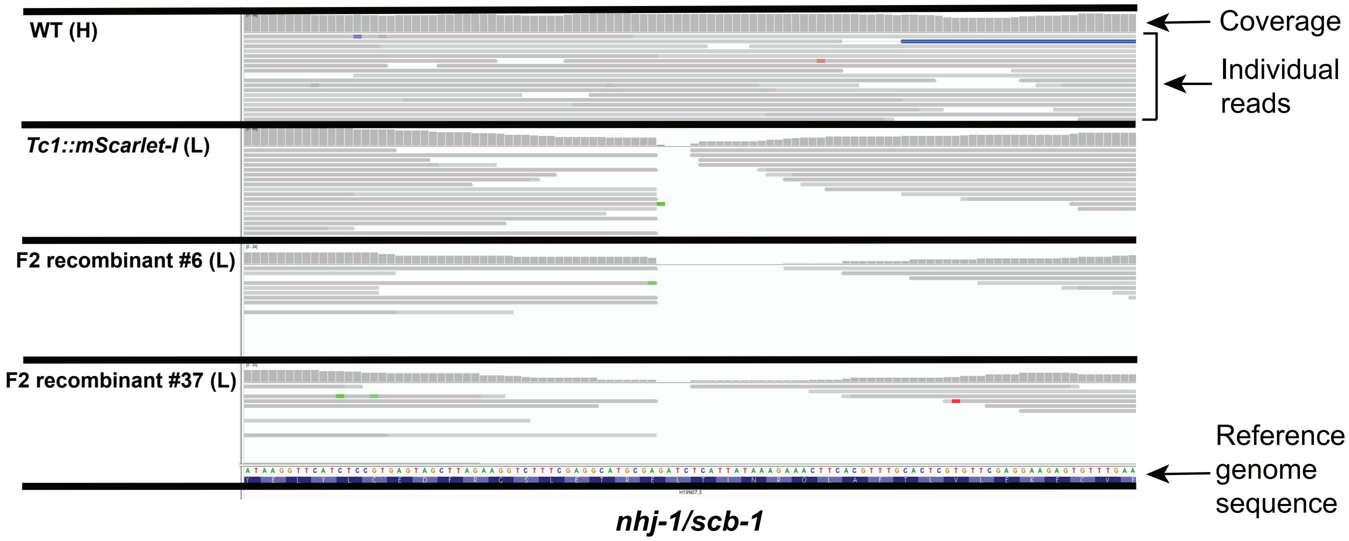

Figure S4. Whole-genome sequencing shows low read coverage in a region of *nhj-1* in L animals. Screenshots from Integrative Genomics Viewer (IGV, https://igv.org/) (Robinson et al. 2011) showing whole-genome sequencing read coverage for WT (H), *Tc1::mScarlet-I* (L), F2 recombinant #6 (L) & #37 (L) derived from the positional mapping cross (shown in Fig. S3b). For each genotype, top vertical gray bars indicate read coverage. Each horizontal gray bar below indicates an individual sequencing read, aligned to the reference genome. In the region shown (which is the coding sequence region of *nhj-1* gene), note the absence of sequence coverage in L but not H animals.

a

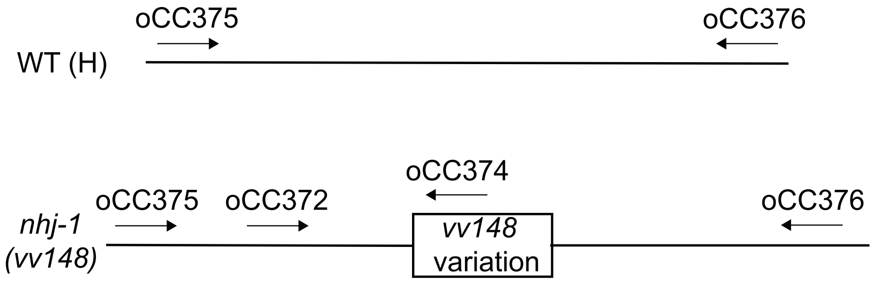

Primer	Sequence (5’-3’)	Reference
oCC372	TTGTGTTGAAACTGTACCGTCT	Vujin et al., 2020
oCC374	TAATAATATTTTTAATAAATAATAGTAATAT	Vujin et al., 2020
oCC375	GTTGGCCAACCTCTTGC	
oCC376	CCAATACGATCTGGCTGATTC	

b

PCR genotyping protocol with Phusion High-Fidelity DNA polymerase (New England Biolabs)

	Primer pair oCC375 & 376	Primer pair oCC372 & 374
Initial denaturation	98°C 3 min.	98°C 3 min.
Denaturation	98°C 10 sec. (35 cycles)	98°C 10 sec. (37 cycles)
Annealing	60°C 20 sec. (35 cycles)	52°C 20 sec. (37 cycles)
Extension	72°C 2 min. 30 sec. (35 cycles)	72°C 1 min. (37 cycles)
Final extension	72°C 5 min.	72°C 5 min.

Expected DNA band sizes from genotyping PCR

Primer pair	Genotype: WT (H)	Genotype: *nhj-1 (vv148)*
oCC375 & oCC376	1972 bp	no band
oCC372 & oCC374	no band	~500 bp

c

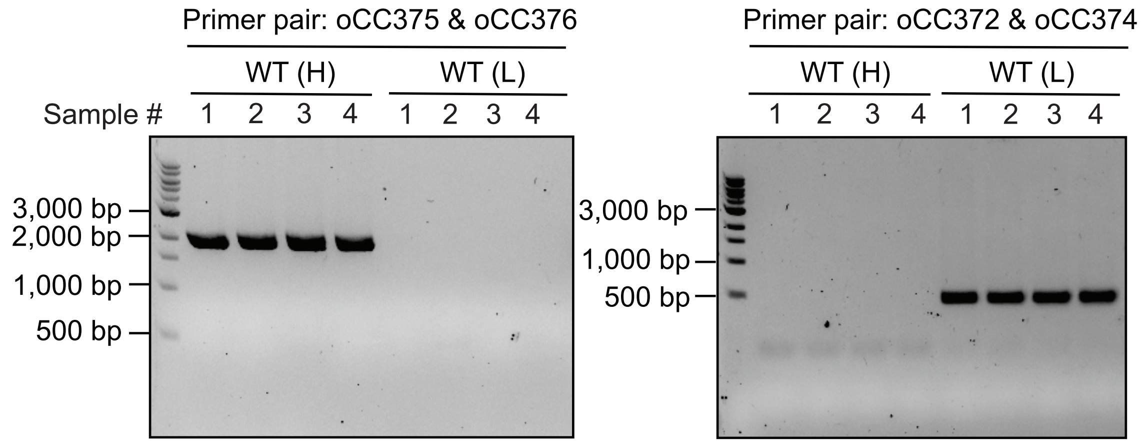

Figure S5. Genotyping analysis shows *nhj-1(vv148)* is present in WT (L). (a) *nhj-1(vv148)* genotyping primers: Primers oCC375 and oCC376 anneal on either side of the *vv148* variation, and will amplify only if *vv148* is not present, due to PCR reaction conditions **(b)**. Primer oCC374 lies within the *vv148* variation (Vujin et al. 2020), and will only amplify with oCC372 if *vv148* is present. **(b)** (Top) PCR cycling conditions for *nhj-1(vv148)* genotyping. (Bottom) Expected PCR band sizes for WT (H) and *nhj-1(vv148)* genotypes. bp, base pairs. **(c)** PCR products using indicated primers run on an agarose gel and stained with ethidium bromide. Sample numbers indicate biological replicates of indicated genotypes.

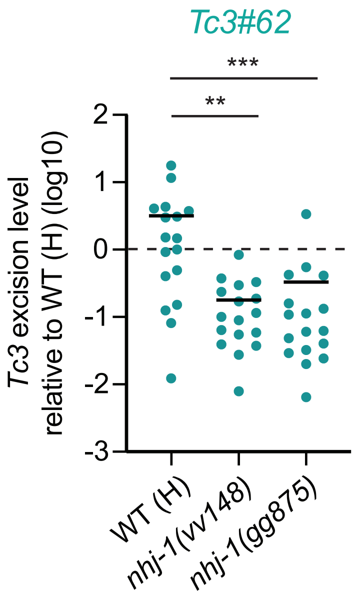

Figure S6. NHJ-1 is a general enhancer of *Tc1/Mariner* class DNA transposon excision. Quantification of *Tc3#62* excision levels by qPCR for indicated genotypes. Data are represented as log10 fold change relative to the mean level of *Tc3#62* excision in WT (H) animals. n= 17. Bars show mean values. **, p≤ 0.01; ***, p≤0.001 (Kruskal-Wallis test with Dunn's test for multiple comparisons). *Tc3#62*, WBTransposon00000062.

# of somatic mScarlet-I events (*Tc1::mScarlet-I*)	
#animals scored	1333
#animals with 1(+) cell	233
#animals with 2(+) cell	77
#animals with >2(+) cell	19

Figure S7. Somatic *Tc1* excision occurs mostly in post-mitotic cells. Somatic *Tc1* excision events in *Tc1::mScarlet-I* animals subcategorized by the number of somatic cells expressing mScarlet-I in all animals possessing one or more cells expressing mScarlet-I. mScarlet-I fluorescence in somatic cells is indicated as (+). Note, 15 of 233 animals listed under "#animals with 1(+) cell" had (+) signal in two neighboring gut nuclei. These were categorized as 1(+) cell animals as this signal likely results from a single *Tc1* excision event that occurred before intestinal cell binucleation, which occurs at the L1 stage (Hedgecock and White 1985).

Supplementary Material References

Hedgecock EM, White JG. 1985. Polyploid tissues in the nematode Caenorhabditis elegans. Dev Biol. 107(1):128–133.

Robinson JT, Thorvaldsdóttir H, Winckler W, Guttman M, Lander ES, Getz G, Mesirov JP. 2011. Integrative genomics viewer. Nat Biotechnol. 29(1):24–26.

Vujin A, Jones SJ, Zetka M. 2020. NHJ-1 Is Required for Canonical Nonhomologous End Joining in Caenorhabditis elegans. Genetics. 215(3):635–651.

Supplementary Table 1: Strain List

Strain name	Genotype	Description of allele	Note
N2			Wild-type
YY2054	*ggSi55 I*	*ggSi55[eft-3p::Tc1::(NLS)mScarlet-I(NLS)::tbb-2 3'UTR] I*	*Tc1::mScarlet-I* (H)
YY2191	*ggSi41 III*	*ggSi41[rpl-28p::Tc1::(NLS)sfgfp(NLS)::unc-54 3'UTR] III*	*Tc1::sfgfp*
YY1887	*ggSi29 I; nhj-1(vv148) V*	*ggSi29[eft-3p::Tc1::(NLS)mScarlet-I(NLS)::tbb-2 3'UTR] I*	
YY2055	*ggSi56 I*	*ggSi56[eft-3p::Tc1::(NLS)mScarlet-I(NLS)::tbb-2 3'UTR] I*	*Tc1::mScarlet-I* (H) Line 1 in Figure 1e
YY2054	*ggSi55 I*	*ggSi55[eft-3p::Tc1::(NLS)mScarlet-I(NLS)::tbb-2 3'UTR] I*	*Tc1::mScarlet-I* (H) Line 2 in Figure 1e
YY2056	*ggSi57 I*	*ggSi57[eft-3p::Tc1::(NLS)mScarlet-I(NLS)::tbb-2 3'UTR] I*	*Tc1::mScarlet-I* (H) Line 3 in Figure 1e
YY2159	*ggSi55 I; nhj-1(gg875) V*		
YY2192	*ggSi41 III; nhj-1(vv148) V*		
YY2003	*ggSi29 I; glp-1(q224ts) III; nhj-1(vv148) V*		
YY2002	*ggSi29 I; glp-1(q224ts) III*		
YY2053	*nhj-1(vv148) V*		
YY2155	*nhj-1(gg875) V*		
FX1524	*cku-70(tm1524) III*		from *Caenorhabditis* Genetics Center
RB964	*cku-80(ok861) III*		from *Caenorhabditis* Genetics Center
RB873	*lig-4(ok716) III*		from *Caenorhabditis* Genetics Center
VC40641			Million Mutation Project strain
YY2096	*rde-3(ne3370) I; ggSi55 I*		
YY2166	*rde-3(ne3370) I; ggSi55 I; nhj-1(vv148) V*		
YY2162	*rde-3(ne3370) I; ggSi55 I; lig-4(ok716) III*		
JK4605	*glp-1(q224ts) III*		from Kimble lab

Boston area N2 strains: From Kim (line 1), Greer (line 2), Colaicovo (line 3), Heiman (line 4) labs

CGC N2 strain: *C. elegans* var Bristol. Annotated as DR subclone of CB original. From the *Caenorhabditis* Genetics Center.
